# Joint clustering of protein interaction networks through Markov random walk

**DOI:** 10.1186/1752-0509-8-S1-S9

**Published:** 2014-01-24

**Authors:** Yijie Wang, Xiaoning Qian

**Affiliations:** 1 Dept. of Electrical & Computer Engineering, Texas A&M University, College Station, TX, 77843, USA; 2 Dept. of Computer Science & Engineering, University of South Florida, Tampa, FL, 33620, USA; 3 Dept. of Pediatrics, College of Medicine, University of South Florida, Tampa, FL, 33620, USA

**Keywords:** Joint Clustering Algorithm, Protein Protein Interaction Networks, Markov Random Walk

## Abstract

Biological networks obtained by high-throughput profiling or human curation are typically noisy. For functional module identification, single network clustering algorithms may not yield accurate and robust results. In order to borrow information across multiple sources to alleviate such problems due to data quality, we propose a new joint network clustering algorithm ASModel in this paper. We construct an integrated network to combine network topological information based on protein-protein interaction (PPI) datasets and homological information introduced by constituent similarity between proteins across networks. A novel random walk strategy on the integrated network is developed for joint network clustering and an optimization problem is formulated by searching for low conductance sets defined on the derived transition matrix of the random walk, which fuses both topology and homology information. The optimization problem of joint clustering is solved by a derived spectral clustering algorithm. Network clustering using several state-of-the-art algorithms has been implemented to both PPI networks within the same species (two yeast PPI networks and two human PPI networks) and those from different species (a yeast PPI network and a human PPI network). Experimental results demonstrate that ASModel outperforms the existing single network clustering algorithms as well as another recent joint clustering algorithm in terms of complex prediction and Gene Ontology (GO) enrichment analysis.

## Introduction

Over the past decade, one goal of systems biology is to understand how different molecules work together to maintain cellular functionalities [1,2]. It is now a common belief that many complex diseases including cancer are due to systems impairments caused by not only single genetic mutations but also disruption of molecular interactions under different situations, which have been conjectured to be the probable sources of disease heterogeneity as well as treatment response heterogeneity [3-5]. Hence, by analyzing large-scale gene expression profiles and protein-protein interaction (PPI) data, computational methods may help us to have a better understanding of biological pathways and cellular organization and thereafter their relationships to diseases as well as potential drug responses [1,2]. One way to investigate these large-scale data is to analyze them in the framework of network analysis [2]. In this paper, we focus on the analysis of PPI networks. We are interested in network clustering to divide the given network into small parts, which can be considered as potential functional modules or pathways [6-8] since biological functions are carried by groups of genes and proteins in a coordinated way [9,10].

There are many existing algorithms for clustering single PPI networks. Normalized cut (NCut) method [11] aims to partition the network based on a novel global criterion, which focuses on the contrast between the total dissimilarity across different clusters and the total similarity within clusters based on network topology. The formulation of NCut is equivalent to finding low conductance sets on the transition matrix of the Markov random walk on the network to analyze [12,13]. Markov CLustering algorithm (MCL) [14] detects clusters based on stochastic flow simulation, which has been proven to be effective at clustering biological networks. Recently, an enhanced version of MCL--Regularized MCL (RMCL) [15,16]--has been proposed to penalize large clusters at each iteration of MCL to obtain more balanced clusters and it has been shown to have better performance to identify clusters with potential functional specificity.

However, it is well known that the current public PPI datasets are quite noisy and there exist both false positive and false negative interactions due to different technical reasons [17]. Therefore, clustering simply based on one network constructed from a single data source may not be able to yield robust and accurate results. We may need to appropriately integrate multiple information sources to repress the noise in existing PPI datasets by borrowing strengths from each other. AlignNemo [18] is one of such recent efforts, which detects network clusters on an alignment network of two given PPI networks. AlignNemo takes into account not only the network topology from two PPI networks but also the homology information between proteins across two networks. However, based on the reported experiments and our empirical findings, AlignNemo has low clustering coverage because the alignment network is constructed based on only similar proteins by their sequence similarity and those proteins that do not appear in the alignment network are never considered for clustering.

In this paper, we propose a joint clustering algorithm based on a new Markov random walk on an integrated network, which is constructed by integrating protein-protein interactions in given PPI networks as well as homological interactions introduced by sequence similarity between proteins across networks. A novel alternative random walk strategy is proposed on the integrated network with the transition matrix integrating both topology and homology information. We formulate the joint clustering problem as searching for low conductance sets defined by this transition matrix. We then derive an approximate spectral solution algorithm for joint network clustering.

The organization of the rest of the paper is as follows: In section 2, we introduce the construction of the integrated network, the new random walk strategy, our final optimization problem formulation and the spectral algorithm for joint clustering. Section 3 contains experimental results on clustering two PPI networks within the same species (two yeast PPI networks and two human PPI networks, respectively) as well as those from different species (one yeast and one human PPI networks). Our experimental results demonstrate that our joint clustering algorithm, which we call it ASModel, outperforms the state-of-the-art single network clustering algorithms as well as AlignNemo [18] in terms of both protein complex prediction and Gene Ontology (GO) enrichment analysis [19]. Finally, we draw our conclusions in section 4.

## Methodology

### Terminology

Let G=(U,D) and H=(V,E)be two PPI networks, where  U and  V are node sets representing *N*_1 _and *N*_2 _proteins in two networks, respectively; and  D and  E denote edges corresponding to respective protein-protein interactions. We assume that  G and  H are connected networks, whose topology structures can be mathematically captured by their corresponding adjacency matrices *A*_1 _and *A*_2_:

(1) $$A_{\text{1}}(i,j)=\{\begin{array}{cc} 1 & (u_{i},u_{j})\in D,\;i\neq j;\\ 0 & otherwise. \end{array}\;A_{\text{2}}(i,j)=\{\begin{array}{cc} 1 & (v_{i},v_{j})\in ℰ,\;i\neq j;\\ 0 & otherwise. \end{array}$$

where *u_i_*, *u_j _*∈  U and *v_i_*, *v_j _*∈  V and we first ignore self-loops in PPI networks. Suppose some of the proteins in  U and  V are known *a priori *to be similar to each other by some criteria, such as their constituent or functional similarity. For example, we compute protein sequence similarity based on the normalized BLAST bit score [20] in this paper so that the latter performance evaluation in our experiments based on curated functional annotations is as unbiased as possible. In a similarity matrix *S*_12_, each element *S*_12_(*u_i_*, *v_j_*) records the similarity between proteins *u_i _*∈  U and *v_j _*∈  V:

(2) $$S_{12}\left(u_{i},v_{j}\right)=\frac{\text{BLAST}\left(u_{i},v_{j}\right)}{\sqrt{\text{BLAST}\left(u_{i},u_{j}\right)}\times \sqrt{\text{BLAST}\left(v_{j},v_{j}\right)}}$$

where BLAST(*u_i_*, *v_j_*) stands for the bit score of sequence similarity between proteins *u_i _*and *v_j _*by BLAST [20]. Based on (2), we note that *S*_12_(*u_i_*, *v_j_*) is in the range [0, 1].

### Integrated network

**Figure 1:**
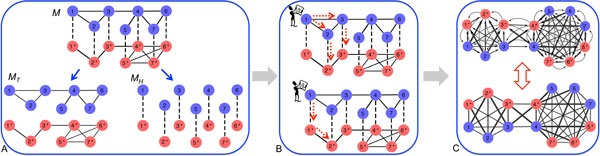
**Illustration of our proposed joint clustering algorithm**. A. Construction of the integrated network. B. Random walk strategy. C. Equivalence between a directed network (transition matrix *P *) and a symmetric undirected network (transition matrix $\bar{P}$).

In order to jointly cluster two PPI networks, we first define a new integrated network $M=\left(W,E_{T},E_{H}\right).$ The set of nodes  Win this integrated network is the union of proteins in two PPI networks (W=U∪V).The integrated network  M has two types of interactions, where $E_{T}$ represents the union of the sets of protein-protein interactions within the PPI networks $\left(E_{T}=D\cup E\right)$ and $E_{H}$ are new "interactions" across two PPI networks introduced by the homological similarity *S*_12_. One example of an integrated network is illustrated in Figure 1A. In this example,  W contains all the nodes in blue and red colors from two respective networks. The solid edges indicate the interactions in $E_{T}$ and the dashed edges represent the interactions in $E_{H}$.

The integrated network combines both the topology information within two PPI networks and the homology information across two PPI networks. Therefore,  M can be considered as the integration of two networks $M_{T}=\left(W,E_{T}\right)$ and $M_{H}=\left(W,E_{H}\right)$, which share the same set of nodes  W. $M_{T}$ is the network carrying the topology information within two PPI networks, whose adjacency matrix can be represented as follows:

(3) $$A={\left[\begin{array}{cc} A_{1} & 0\\ 0 & A_{2} \end{array}\right]}_{N\times N}$$

where *N *= *N*_1 _+ *N*_2_. $M_{H}$is the network containing the homology information across two networks, whose adjacency matrix can be represented as

(4) $$S={\left[\begin{array}{cc} 0 & S_{12}\\ S_{12}^{T} & 0 \end{array}\right]}_{N\times N}.$$

The examples of $M_{T}$ and $M_{H}$ are also illustrated in Figure 1A.

### Random walk strategy on the integrated network

As shown in the previous section, the integrated network contains both topology and homology information represented in two sets of edges. In order to bring strengths from each other to improve the clustering performance in individual networks, we propose a random walk strategy on the integrated network  M to integrate all information sources. To make use of both topology and homology information, we require the random walker must walk through topological and homological interactions ($E_{T}$ and $E_{H}$) in an alternative order. However, as shown in Figure 1B, the random walker can either first walk by $M_{T}$ then on the network $M_{H}$ or first walk on $M_{H}$ then on $M_{T}$. For the first type of random walk illustrated in Figure 1B, the transition matrix $P_{A\bar{S}}$ can be calculated as

(5) $$P_{A\bar{S}}=P_{A}\times P_{\bar{S}}$$

where $P_{A}=D_{A}^{-1}A$ and $P_{\bar{S}}=D_{\bar{S}}^{-1}\bar{S}$. The matrix *D_A _*is a diagonal matrix with the degree of each node on its diagonal elements. $\bar{S}=S+I_{N\times N}$ is the adjacency matrix of network $M_{H}$ with self-loops indicating self similarity of proteins. $D_{\bar{S}}$ is the corresponding diagonal matrix with $D_{\bar{S}}\left(i,i\right)=\sum_{j}\bar{S}\left(i,j\right)$, where *i*, *j *∈ {1, 2, ..., *N*} are new node indices in the integrated network and $\bar{S}\left(i,j\right)>0$ when *i*, *j *indicate proteins from different PPI networks. Again, $\bar{S}\left(i,i\right)=1$ for self similarity. Further-more, we find that *P_A _*is the transition matrix of the random walk on $M_{T}$ and $P_{\bar{S}}$ is the transition matrix of the random walk on $M_{H}$ including self-loops.

For the second type of random walk illustrated in Figure 1B, we can similarly compute the transition matrix

(6) $$P_{SĀ}=P_{S}\times P_{Ā}$$

where $P_{S}=D_{S}^{-1}S$ and $P_{Ā}=D_{Ā}^{-1}Ā$. Here, *D_S _*is a diagonal matrix with $D_{S}\left(i,i\right)=\sum_{j}S\left(i,j\right)$. Here,  Ā is the adjacency matrix of $M_{T}$ with self-loops to allow for the possibility of random walker staying at the current node. $D_{Ā}$ is the corresponding diagonal matrix with the node degree in  Ā on its diagonal. *P_S _*is the transition matrix of the random walk on $M_{H}$ and $P_{Ā}$ is the transition matrix of the random walk on $M_{T}$ including self-loops.

We further assume that the probability of taking the first type of random walk should be the same as going with the second type of random walk. Therefore, our final transition matrix for the new random walk strategy can be represented by

(7) $$P=\frac{1}{2}P_{A\bar{S}}+\frac{1}{2}P_{SĀ}$$

### Searching for low conductance sets based on *P*

In $M_{T}$, proteins with topological interactions $E_{T}$ are likely to participate in similar cellular functions. Also, proteins with larger homological interactions $E_{H}$ in $M_{H}$ are more probable to be functionally similar. Because the random walk on the integrated network considers both types of interactions, each element *P *(*i*, *j*) of the corresponding transition matrix can be understood as the probability that proteins *i *and *j *have similar functions as these proteins are more likely to reach each other with a larger *P *(*i*, *j*). Based on this, we can make use of the concept of the conductance defined on the Markov chain to identify clusters based on *P *[11,21] by searching for low conductance sets.

Similarly as done in [11,21], we can formulate the optimization problem for joint network clustering:

(8) $$\text{min}\overset{k}{\underset{h=1}{\sum }}\Phi_{P}\left(C_{h},{\bar{C}}_{h}\right)\;\;\;\;\;\;\;\;\;s.t.\overset{k}{\underset{h=1}{⋃}}C_{h}=W;\;\;\;\;C_{h}\cap C_{l}=\emptyset ,\forall h\neq l.$$

where $\Phi_{P}\left(C_{h},{\bar{C}}_{h}\right)$ is the defined conductance of node subset *C_h _*to the rest of the network ${\bar{C}}_{h}$; and *k *is the number of desired subsets as final network clusters. The conductance $\Phi_{P}\left(C_{h},{\bar{C}}_{h}\right)$ can be computed as

(9) $$\Phi_{P}\left(C_{h},{\bar{C}}_{h}\right)=\frac{\sum_{i\in C_{h},j\in {\bar{C}}_{h}}\pi_{i}P\left(i,j\right)}{\sum_{i\in C_{h}}\pi_{i}},C_{h}\cup {\bar{C}}_{h}=W,$$

where  π is the stationary distribution of the corresponding Markov random walk on the integrated network and $P^{T}\pi =\pi$.

The goal now is to find *k *low conductance sets defined by *P*. As in [21], we find that if we consider *P *as the transition matrix for a directed graph and try to find *k *low conductance sets based on (8), it is in fact equivalent to find *k *low conductance sets on an undirected graph with another transition matrix $\bar{P}$:

(10) $$\bar{P}=\frac{\pi P+P^{T}\pi }{2}.$$

Due to the equivalence, our optimization formulation for finding *k *low conductance sets can be formulated finally as

(11) $$\begin{array}{c} \text{max}\;\;\;\;\;\;\;\;\;\;trace\left(\frac{X^{T}\bar{P}X}{X^{T}D_{\bar{P}}X}\right)\\ \;\;s.t.\;\;\;\;\;\;\;\;\;X1_{k}=1_{N},x_{i\ell }\in \left\{0,1\right\}, \end{array}$$

where $D_{\bar{P}}$ is a diagonal matrix with $D_{\bar{P}}\left(i,i\right)=\sum_{j}\bar{P}\left(i,j\right)$; *X *is a *N × k *assignment matrix whose element *x_iℓ _*denotes whether node *i *belongs to cluster *ℓ*; 1*_k _*and 1*_N _*are all one vectors with *k *and *N *elements, respectively. Here, equations (8) and (11) have been proven to be equivalent previously in [21]. We can derive a spectral method to solve the above problem based on [12]. The directed network with *P *and its equivalent undirected network with $\bar{P}$ are illustrated in Figure 1C.

### Joint Clustering Algorithm (ASModel)

Our joint clustering algorithm can be summarized into three steps which are illustrated in Figure 1. The first step is to construct the integrated network  M. The second step is to compute the transition matrix *P *based on the alternative random walk strategy in (7). The final step is to find low conductance sets on the equivalent network and apply the spectral method to solve the optimization problem. Algorithm 1 provides the pseudo code for ASModel.

**Algorithm 1**. ASModel for Joint Network Clustering

**Input: **Adjacency matrices *A*_1 _and *A*_2_, Sequence similarity matrix *S*_12_, and the number of desired clusters *k*

**Output: **Cluster assignment matrix *X*

1. Construct the integrated network  M and compute *A *and *S*;

2. Compute the transition matrix *P *based on the random walk strategy using (7);

3. Obtain the equivalent adjacency matrix $\bar{P}$ which has the same low conductance sets as *P*;

4. Using the spectral algorithm to find *k *low conductance sets by $\bar{P}$ from (11) [12].

## Experiments

### Algorithms, data, and metrics

We compare our joint clustering algorithm ASModel to NCut [11], MCL [14], RMCL [15,16], and AlignNemo [18]. Among the selected algorithms for performance comparison, AlignNemo [18] is a recently proposed protein complex detection algorithm, which also takes into account the homology and topology information from two PPI networks. NCut is equivalent to searching for low conductance sets by the transition matrix defined directly based on the given single network. Therefore, comparing with NCut aims to show that finding low conductance sets on the integrated network by our new ASModel is superior to separately finding similar low conductance sets on individual networks. MCL and RMCL are two state-of-the-art algorithms which have been proven effective on analyzing biological networks. Comparing with them can further demonstrate that our joint clustering algorithm ASModel can achieve better performances than clustering single networks separately. Both NCut and ASModel have one input parameter, which is the number of clusters *k*. We sample *k *in [100, 3000] with an interval of 100 and report the best results. MCL also has one parameter, the inflation number. We similarly search for the best performing value from 1.2 to 5.0 with an interval of 0.1. For RMCL, we adopt the parameters suggested in [15,16]. AlignNemo is a heuristic algorithm without any tuning parameters [18] and we directly implement the provided algorithm in our experiments.

In addition to evaluating joint clustering by ASModel using synthetic networks, we evaluate the performances of ASModel, NCut, MCL, RMCL, and AlignNemo on public PPI datasets for *S. cerevisiae *(budding yeast) and *H. sapiens *(human). For *S. cerevisiae*, *Sce*DIP and *Sce*BGS are two extracted PPI networks from the Database of Interacting Proteins (DIP) [22] and BioGRID [23], respectively. For *H. sapiens*, *Hsa*HPRD and *Hsa*PIPs are corresponding PPI networks derived from Human Protein Reference Database (HPRD) [24] and the PIPs dataset [25]. The details of each PPI network are given in Table 1.

**Table 1:** Information of four real-world PPI networks.

Network	#. nodes	#. edges	SGD	CORUM	*|GO|*
*Sce*DIP	4980	22076	305	--	956
*Sce*BGS	5640	59748	306	--	1005
*Hsa*HPRD	9269	36917	--	1294	4755
*Hsa*PIPs	5226	37024	--	1193	4560

In order to access the performance of the competing algorithms, we first implement complex prediction to assess the quality of clustering results by evaluating the agreement of the clusters found by each method with curated protein complex standards. SGD [26] and CORUM [27] complexes are considered as the golden standards for complex prediction for yeast and human PPI networks, respectively. We then implement the GO enrichment analysis for further validation on function predicting performance from clustering results. In order to focus on more specific cellular functions, we use specific GO terms with information content (*IC*) larger or equal to 2, filtered out from all three domains: biological process, molecular function, and cellular component. The information content of a GO term *g *is defined as:

(12) $$IC\left(g\right)=-log\left(\left|g\left|/\right|root\right|\right),$$

where *|g| *and *|root| *are the number of proteins in GO term *g *and the number of proteins in its corresponding GO category. The information of reference complex datasets and GO terms is also provided in Table 1.

We adopt the widely used F-measure [28] to evaluate the performance for complex prediction. F-measure is the harmonic mean of precision and recall: *F *= 2 × precision × recall/(precision + recall), where precision and recall are defined as follows:

(13) $$\text{precision}=\frac{\left|\left\{C_{i}\in C|NA\left(C_{i},R_{j}\right)>0.\text{25},\exists R_{j}\in R\right\}\right|}{\left|\text{C}\right|};$$

(14) $$\text{recall}=\frac{\left|\left\{R_{i}\in R|NA\left(C_{i},R_{j}\right)>0.\text{25},\exists C_{i}\in C\right\}\right|}{\left|R\right|},$$

where *C *= {*C*_1_, *C*_2_, ..., *C_k_*} are the identified clusters by different algorithms and *R *= {*R*_1_, *R*_2_, ..., *R_l_*} denote the corresponding reference complex sets. The neighbor affinity $NA\left(C_{i},R_{j}\right)=\frac{|C_{i}\cap R_{j}{|}^{2}}{\left|C_{i}\left|\times \right|R_{j}\right|}$ measures the overlap between the predicted complex *C_i _*and the reference complex *R_j_*.

To evaluate the performance of GO enrichment analysis, we compute the p-value and the number of enriched GO terms from clustering results. Suppose that the whole network has *N *proteins with *M *proteins annotated with one GO term and the detected cluster has *n *proteins with *m *proteins annotated with the same GO term. The p-value of the cluster with respect to that GO term can be calculated as [29]

(15) $$\text{p-value = }\sum_{i=m}^{n}\frac{\left(\begin{array}{c} m\\ i \end{array}\right)\left(\begin{array}{c} N-M\\ N-i \end{array}\right)}{\left(\begin{array}{c} N\\ n \end{array}\right)}$$

We choose the lowest p-value of all enriched GO terms in the derived cluster as its final p-value. A GO term is enriched when the p-value of any cluster corresponding to this GO term is less than 1*e*-3.

### Synthetic networks

We first evaluate and compare the clustering performance of our proposed ASModel with the performances of running random walk on individual networks as well as running the random walk directly on integrated networks with both interactions within networks and similarity across networks. The goal of this set of experiments is to demonstrate that not only joint clustering performs better than clustering individual networks by NCut, but also our proposed ASModel can achieve better performance than the normal random walk on the integrated work using the same set of integrated information.

**Figure 2:**
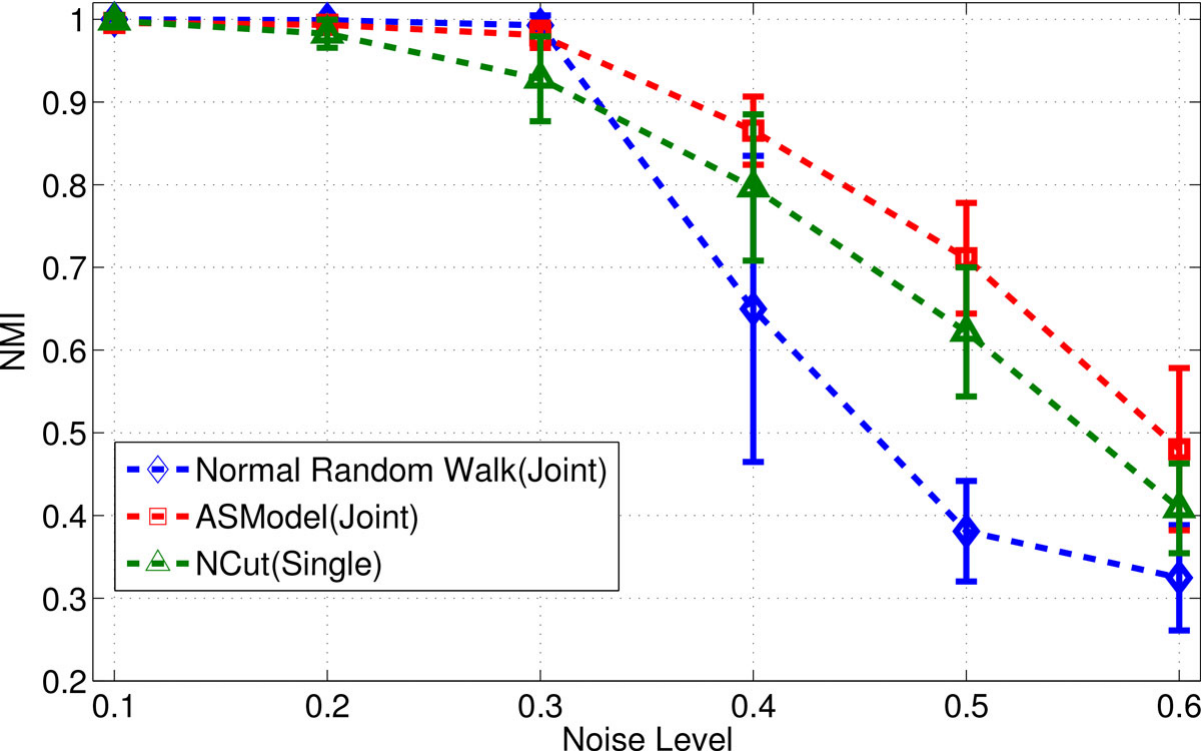
**Performance comparison on synthetic networks for random walk algorithms**.

We first generate two noise-free individual networks. The first network has 4 modules, each of which has 24 nodes. The second network also has 4 modules, each of which has 36 nodes. The edge density in each modules of both individual networks are 0.5. We further assign the nodes in the corresponding pairs of modules across two networks as potential "orthologous" node pairs. In this set of experiments, we set the similarity density of nodes within the corresponding modules to 0.2, meaning that 20% node pairs among all possible node pairs within the corresponding modules are randomly assigned to be similar to each other. We further add noise to both the interactions within individual networks and the node similarity across networks by randomly permuting a certain percentage of edges (both interaction and similarity) by Maslov-Sneppen procedure [30], which enables the performance evaluation at different noise levels. As we have the ground truth of modular structures in synthetic networks, we use the normalized mutual information (NMI) [31] as the evaluation criterion. We have generated 30 pairs of synthetic networks for each noise level and the average NMI values and their standard deviations of clustering results from three different random walk schema are shown in Figure 2 for the performance comparison. For joint clustering algorithms, we find that when the noise level is low, ASModel and normal random walk are competitive with each other. However, with the increasing noise level, ASModel clearly outperforms normal random walk. Comparing ASModel with single network clustering algorithm NCut, we observe that ASModel is also superior to NCut, indicating that joint clustering does achieve better clustering performance than clustering individual networks separately.

### Joint clustering of PPI networks within the same species

In this section, we first jointly cluster two PPI networks from the same species to demonstrate the effectiveness of our ASModel. Through applying ASModel, we expect that each PPI network can borrow strengths from the other PPI network to enhance the clustering performance.

#### Joint clustering of the SceDIP and SceBGS PPI networks

##### Complex prediction

**Figure 3:**
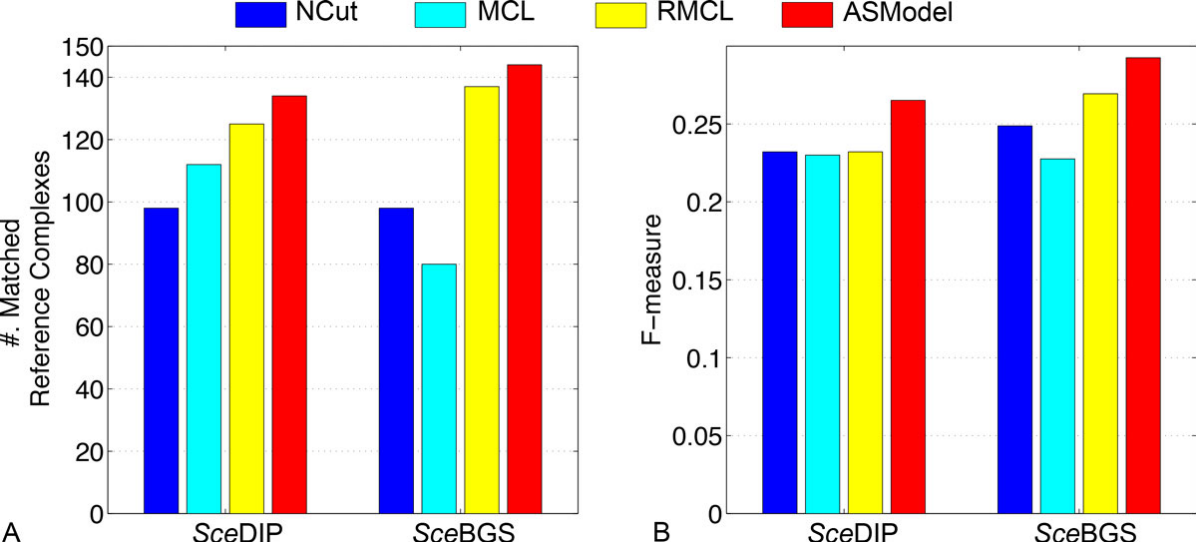
**Performance comparison of competing algorithms for complex prediction in both yeast PPI networks**. A. Comparison on the number of matched reference complexes. B. Comparison on the F-measure.

For the *Sce*DIP and *Sce*BGS networks, we report the performance of ASModel, NCut, MCL, RMCL, and AlignNemo on complex prediction in terms of the number of matched reference complexes and F-measure. The detailed information such as the number of clusters (cluster size ≥ 2) and the coverage is listed in Table 2. Figures 3A and 3B show the comparison results for the number of matched reference complexes and F-measure, respectively. As illustrated in Figure 3, ASModel detects the largest number of matched reference complexes and achieves the highest F-measure for both networks, which is substantially better than the results obtained by individual clustering using all the other single network clustering algorithms. Although AlignNemo also uses both topology and homology information, it is interesting to observe that it does not detect any matched reference complexes in this set of experiments, which in fact is different from the reported results in [18] though different networks were analyzed.

**Table 2:** The information of the derived clusters by all competing algorithms

PPI	Method	NCut	MCL	RMCL	ASModel	ASModel	ASModel
					(DIP+BGS)	(HPRD+PIPs)	(DIP+HPRD)
*Sce*DIP	#. clusters	525	659	814	737	--	702
	coverage	2572	3630	3725	**4537**	--	4425

*Sce*BGS	#. clusters	414	338	772	704	--	--
	coverage	4879	3544	**5210**	5169	--	--

*Hsa*HPRD	#. clusters	981	1239	1508	--	1113	1231
	coverage	6534	7800	6879	--	8631	**8729**

*Hsa*PIPs	#. clusters	491	576	581	--	560	--
	coverage	**4542**	4134	3966	--	4358	--

One important reason that we have seen different results for protein complex prediction by AlignNemo is that we here use a more strict evaluation criterion to consider that a reference complex *R_j _*is recovered by the identified cluster *C_i _*by clustering algorithms only when *N A*(*C_i_*, *R_j _*) > 0.25. In the original paper of AlignNemo [18], a reference complex is considered to be recovered if at least two of its proteins overlap with a detected cluster, which may introduce the evaluation bias. Imagine that if one cluster contains 10 proteins, with every two belonging to a different reference complex. This evaluation criterion will conclude that five different complexes are recovered by the algorithm but the clustering results may not necessarily be desired. Our obtained results may indicate that the random walk strategy in our ASModel better integrates available information across networks than the heuristic strategy adopted in AlignNemo to discover biologically more meaningful clusters.

##### GO enrichment analysis

**Figure 4:**
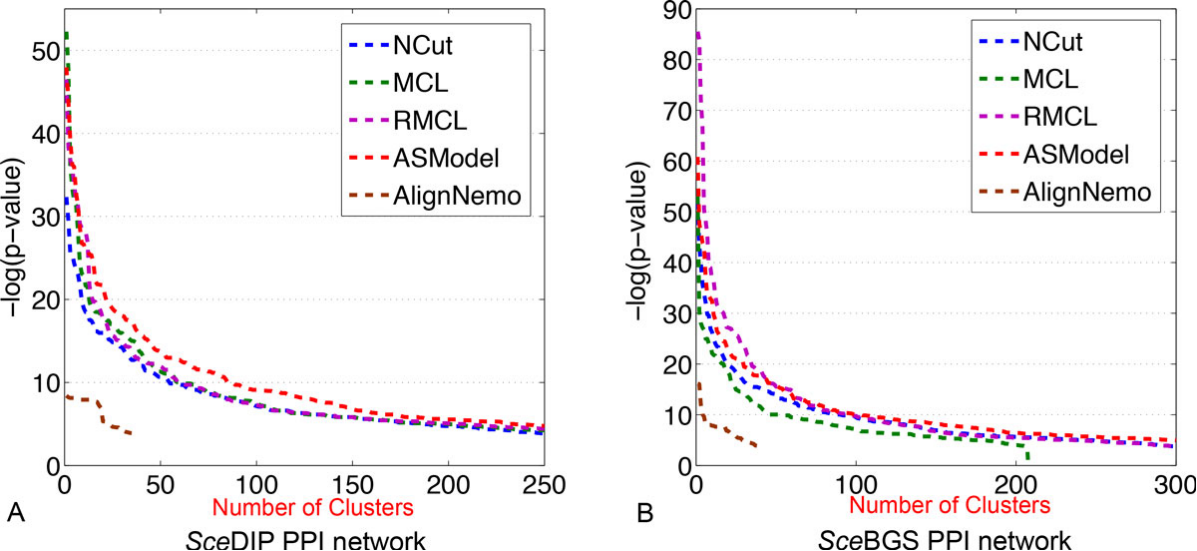
**Performance comparison of competing algorithms for GO enrichment analysis**. A. GO enrichment comparison on the *Sce*DIP network. B. GO enrichment comparison on the *Sce*BGS network.

**Figure 5:**
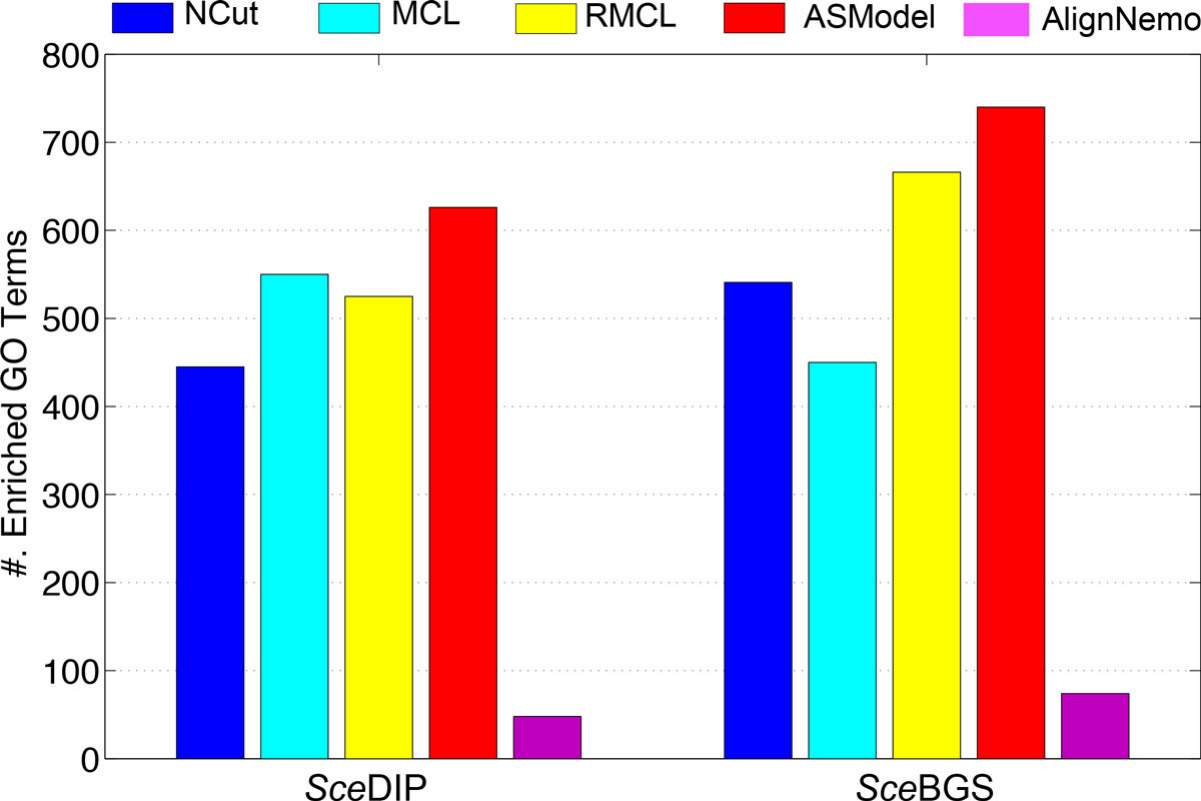
**Comparison on the number of enriched GO terms for all the competing algorithms in two yeast networks**.

GO enrichment analysis has been done based on the detected clusters by ASModel, NCut, MCL, RMCL, and AlignNemo. For each cluster, it may be enriched in multiple GO terms and we choose the lowest p-value as the p-value for the cluster as explained earlier. We first sort the p-values of all clusters in an ascending order and then draw the corresponding monotonically decreasing -*log*(p-value) curves for all the algorithms in Figure 4. As shown in Figure 4A, for the *Sce*DIP PPI network, the curve of ASModel is on top of all the other competing algorithms, which indicates that the clusters detected by joint clustering ASModel are more consistent to the curated GO terms and hence capture the cellular functionalities better. For the *Sce*BGS PPI network from Figure 4B, we find that the curve of RMCL is on top of other algorithms for around the top 80 most significantly enriched clusters. However, when we check more derived clusters, the curve of ASModel is again on top of the other algorithms. Hence, overall, especially when we consider the total number of enriched GO terms shown in Figure 5, functional consistency of the detected clusters is improved by our joint clustering algorithm ASModel as ASModel can identify more enriched GO terms to unearth more biologically meaningful clusters with more significant p-values overall.

In summary, from both complex prediction and GO enrichment analysis, ASModel can achieve more biologically meaningful results. These promising results imply that joint clustering can improve the clustering performance for every individual PPI network when we integrate information from them appropriately.

#### Joint clustering of the HsaHPRD and HsaPIPs networks

##### Complex prediction

**Figure 6:**
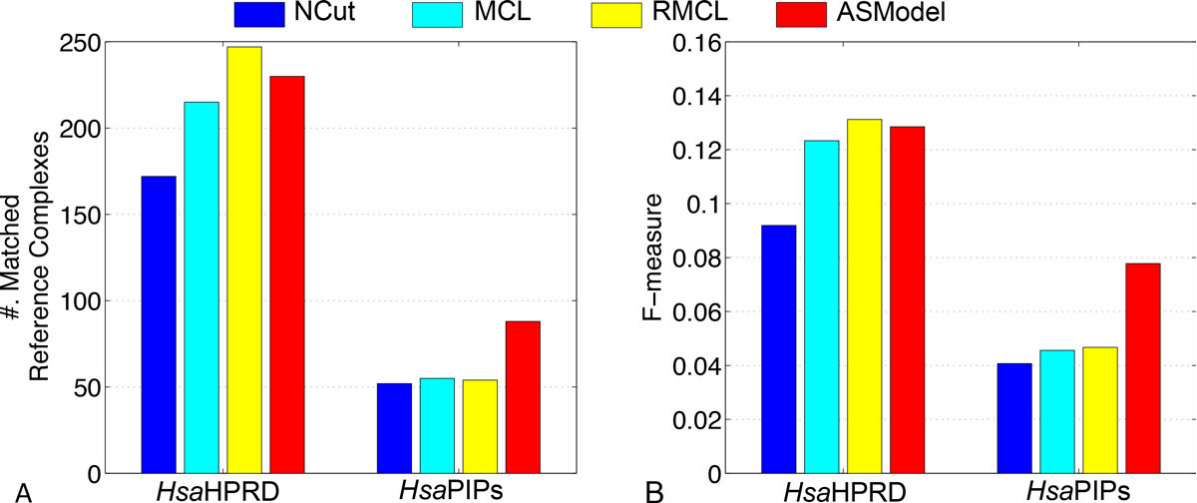
**Performance comparison of competing algorithms for complex prediction in both human PPI networks**. A. Comparison on the number of matched reference complexes. B. Comparison on the F-measure.

Similarly, the results of complex prediction from all the competing algorithms on two human PPI networks are shown in Figure 6. For the *Hsa*HPRD network, we find that RMCL and ASModel detect competitive numbers of reference complexes and achieve competitive F-measures. When we check the *Hsa*PIPs network, Figure 6 shows that ASModel identifies much more matched reference complexes and obtain substantially better F-measure than all the other algorithms. AlignNemo again does not detect any matched reference complexes based on the neighbor affinity metric. The performance of ASModel demonstrates that the clustering of *Hsa*PIPs network does benefit from the information in the *Hsa*HPRD network to achieve the better complex prediction performance. However, the performance on the *Hsa*HPRD network is not influenced much, probably due to the incompleteness of the *Hsa*PIPs dataset.

##### GO enrichment analysis

**Figure 7:**
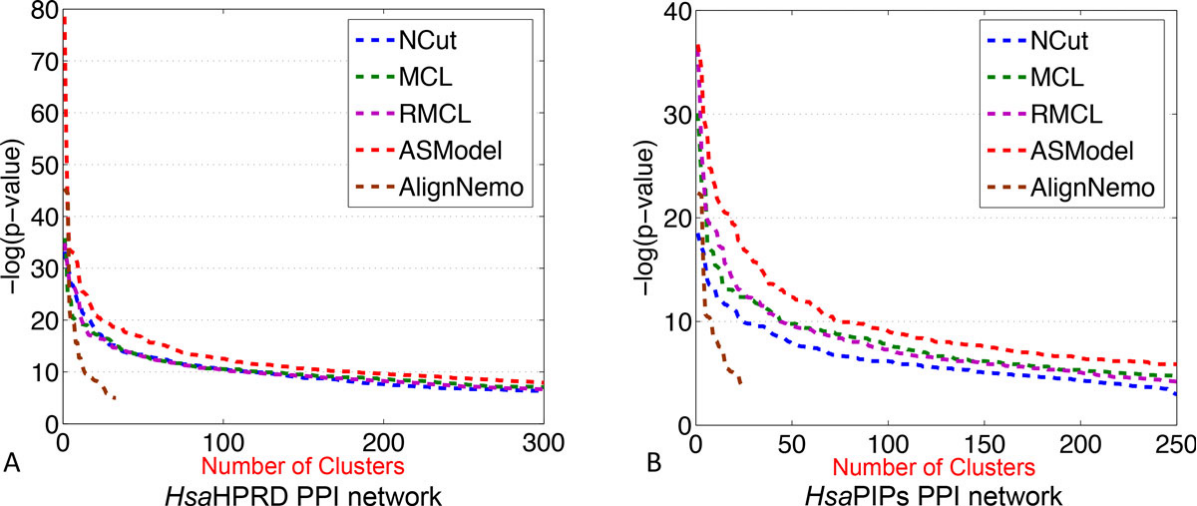
**Performance comparison of competing algorithms for GO enrichment analysis**. A. GO enrichment comparison on the *Hsa*HPRD network. B. GO enrichment comparison on the *Hsa*PIPs network.

**Figure 8:**
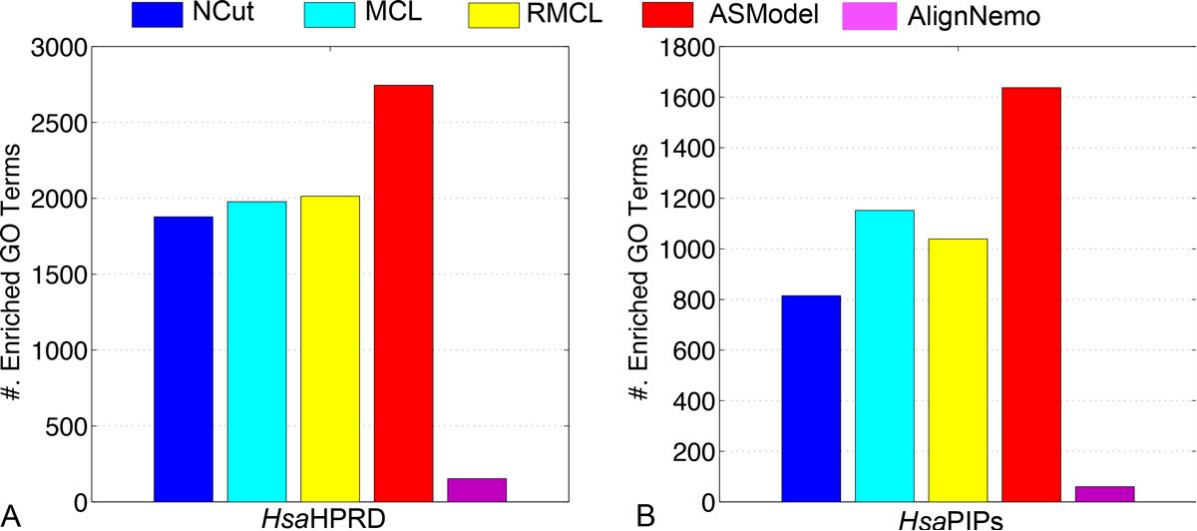
**Comparison on the number of enriched GO terms for all the competing algorithms in two human networks**.

We compare ASModel to NCut, MCL, RMCL, and AlignNemo on GO enrichment analysis by drawing similar *-log*(p-value) curves of the top ranked clusters based on their enrichment significance. From Figure 7, we observe that for both human PPI networks, the curves of ASModel are on top of all the competing algorithms. Furthermore, as shown in Figure 8, we find that ASModel also detects the largest number of enriched GO terms on both networks. The overall performance of GO enrichment analysis further validates that joint clustering significantly enhances the clustering performance for each PPI network.

From these two experiments of joint clustering PPI networks from the same species, we note that ASModel can make full use of topology and homology information to improve the clustering performance for each PPI network.

### Joint clustering of PPI networks from different species

Joint clustering of PPI networks within the same species has been proven to yield promising results. In order to show that ASModel can also improve the clustering performance for PPI networks from different species, we have done the following experiment.

#### Joint clustering with SceDIP and HsaHPRD PPI networks

##### Complex prediction

**Figure 9:**
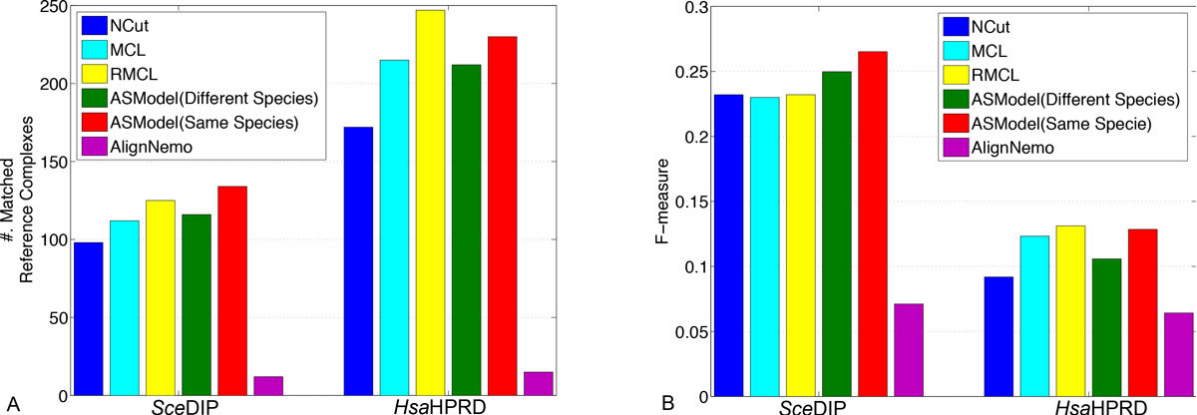
**Performance comparison of competing algorithms for complex prediction in the *Sce*DIP and *Hsa*HPRD network**. A. Comparison on the number of matched reference complexes. B. Comparison on the F-measure. ASModel (Different Species) indicates the results obtained by joint clustering of the *Sce*DIP and *Hsa*HPRD PPI networks. ASModel (Same Species) indicates the results obtained from joint clustering of the *Sce*DIP and *Sce*BGS networks for yeast and joint clustering of the *Hsa*HPRD and *Hsa*PIPs PPI networks for human, respectively.

We first report the performance for protein complex prediction. For the *Sce*DIP network, we compare the results of joint clustering of the *Sce*DIP and *Hsa*HPRD networks by ASModel, joint clustering of the *Sce*DIP and *Sce*BGS networks by ASModel, as well as results obtained from AignNemo and other single network clustering algorithms. We observe in Figure 9 that joint clustering of the *Sce*DIP and *Sce*BGS networks yields the best F-measure and the largest number of matched reference complexes. However, joint clustering of the *Sce*DIP and *Hsa*HPRD networks achieves the second best F-measure and detects competitive numbers of matched reference complexes as RMCL.

For the *Hsa*HPRD network, we compare the results of ASModel obtained from joint clustering of the *Hsa*HPRD and *Hsa*PIPs networks as well as joint clustering of the *Hsa*HPRD and *Sce*DIP PPI networks, AlignNemo, NCut, MCL, and RMCL. The comparison for the number of matched reference complexes and F-measure is given in Figure 9. From the figure, we find that RMCL gets the best performance in terms of these two metrics. ASModel achieves the competitive performance when joint clustering two human networks as shown before. ASModel for two human networks provides better results than jointly analyzing two networks for yeast and human. From this set of experiments, we find that joint clustering two networks within the same species works better than analyzing networks for different species. We in fact expect this because networks within the same species have more shared information, which can be utilized to supplement each other to improve clustering performance. Otherwise, for two networks for different species, joint clustering may not help as much since they may have different cellular constitution and organization due to evolutionary differences.

##### GO Enrichment analysis

**Figure 10:**
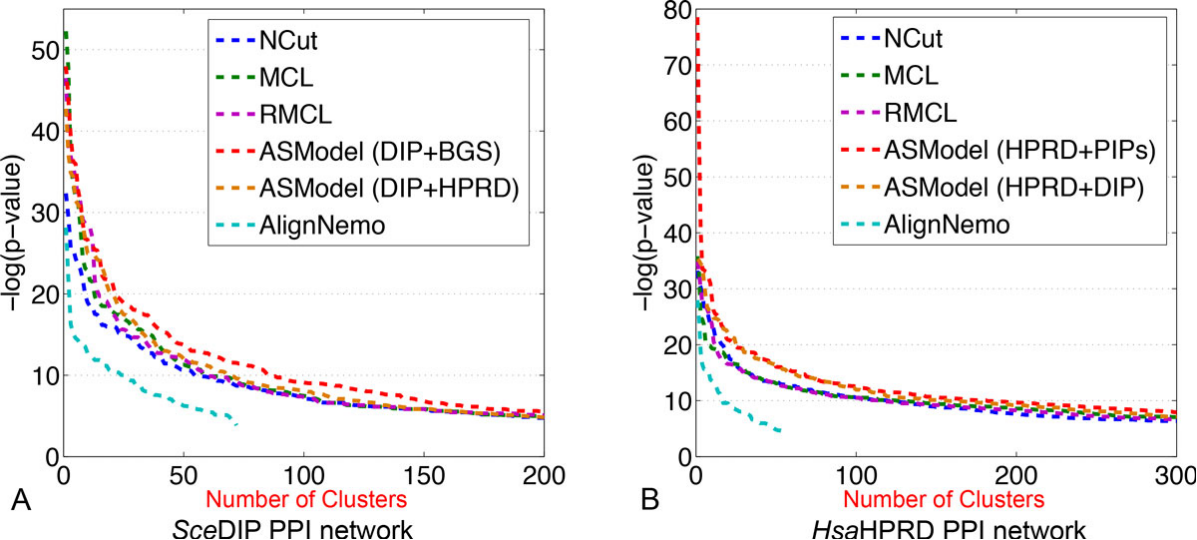
**Performance comparison of competing algorithms for GO enrichment analysis on the *Sce*DIP and *Hsa*HPRD networks**. A. GO enrichment comparison on the *Sce*DIP network. B. GO enrichment comparison on the *Hsa*HPRD network.

**Figure 11:**
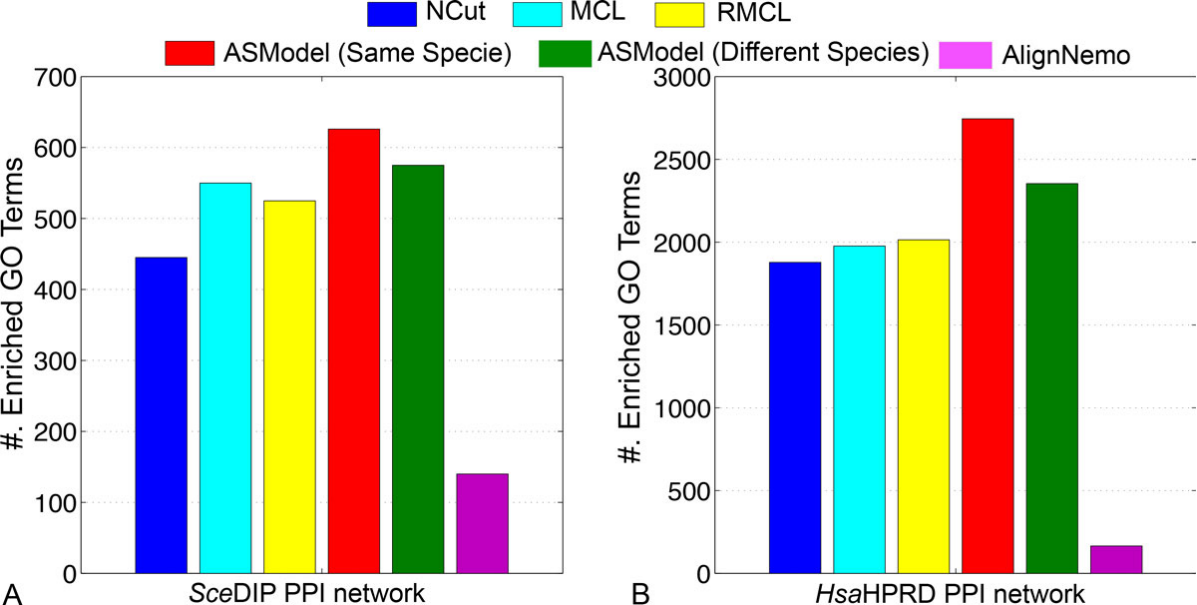
**Comparison on the number of enriched GO terms for all the competing algorithms in the *Sce *DIP and *Hsa*HPRD networks**.

We further illustrate the performance comparison for clustering the *Sce*DIP network in Figure 10A. We note that the curve of ASModel for the *Sce*DIP and *Sce*BGS networks is on top of the curve of ASModel for the *Sce*DIP and *Hsa*HPRD PPI networks. Furthermore, both curves from ASModel are on top of all the other algorithms. With respect to the *Hsa*HPRD PPI networks, we have the same observation that ASModel analyzing PPI networks within the same species is on top of ASModel analyzing networks from different species. Both of them are on top of the others. This further convinces us that joint clustering does improve the clustering performance. In addition, the more information that two PPI networks share, the more enhancement can be achieved by joint clustering. From the comparison of the number of enriched GO terms as shown in Figure 11, we have the same conclusion. ASModel analyzing networks within the same species detects the largest number of enriched GO terms. For analyzing networks from different species, ASModel identifies the second largest number of enriched GO terms among all competing algorithms.

From these experiments, no matter analyzing two PPI networks from the same species or from two different species, our joint clustering algorithm ASModel can achieve better results than analyzing these networks separately using single network clustering algorithms. Furthermore, we find that joint clustering using two PPI networks from the same species achieves more significant performance improvement than using two PPI networks form different species, which coincides with our intuition that we can find more robust and accurate clustering results if we use networks from the same species or species that are phylogenetically close so that the conservation across networks helps to derive more confident clustering results.

## Conclusions and future work

In this paper, we have proposed a joint network clustering algorithm ASModel based on a new alternative random walk strategy. The experimental results based on both complex prediction and GO enrichment analysis demonstrate that using ASModel to joint clustering two PPI networks can achieve better clustering results than single network clustering algorithms and AlignNemo. Furthermore, from comparing with the performances of joint clustering PPI networks within the same species (section 3.2) and those from different species (section 3.3), we find that the more information the PPI networks in the integrated network share, the better the clustering results can be achieved. For our future work, we are collaborating with biologists to explore the potential opportunities using our ASModel to identify biologically meaningful clusters in different species. By carefully investigating recovered clusters, we may have a better understanding of protein functionalities, cellular organization, as well as the underlying signal transduction mechanisms for deriving future systematic intervention strategies.

## Competing interests

The authors declare that they have no competing interests.

## Authors' contributions

Conceived the algorithm: YW, XQ. Implemented the algorithm and performed the experiments: YW. Analyzed the results: YW, XQ. Wrote the paper: YW, XQ.

## Declarations

Publication of this article was funded by the faculty startup fund for XQ from Texas A&M University.

This article has been published as part of *BMC Systems Biology *Volume 8 Supplement 1, 2014: Selected articles from the Twelfth Asia Pacific Bioinformatics Conference (APBC 2014): Systems Biology. The full contents of the supplement are available online at http://www.biomedcentral.com/bmcsystbiol/supplements/8/S1.
